# Alterations in Gray Matter Structural Networks in Amnestic Mild Cognitive Impairment: A Source-Based Morphometry Study

**DOI:** 10.3233/JAD-231196

**Published:** 2024-08-27

**Authors:** Tania M. Setiadi, Jan-Bernard C. Marsman, Sander Martens, Shankar Tumati, Esther M. Opmeer, Fransje E. Reesink, Peter P. De Deyn, Mercedes Atienza, André Aleman, Jose L. Cantero

**Affiliations:** a Department of Biomedical Sciences of Cells & Systems, Cognitive Neuroscience Center, University of Groningen, University Medical Center Groningen, Groningen, The Netherlands; b Neuropsychopharmacology Research Group, Sunnybrook Research Institute, University of Toronto, Toronto, ON, Canada; c Department of Health and Welfare, Windesheim University of Applied Sciences, Zwolle, The Netherlands; d Department of Neurology, University of Groningen, University Medical Center Groningen, Groningen, The Netherlands; e Laboratory of Neurochemistry and Behavior, Experimental Neurobiology Group, University of Antwerp, Antwerp, Belgium; f Laboratory of Functional Neuroscience, Pablo de Olavide University, Seville, Spain; g CIBER de Enfermedades Neurodegenerativas (CIBERNED), Instituto de Salud Carlos III, Madrid, Spain; h Department of Psychology, University of Groningen, University Medical Center Groningen, Groningen, The Netherlands

**Keywords:** Alzheimer’s disease, amnestic mild cognitive impairment, magnetic resonance imaging, source-based morphometry, structural network

## Abstract

**Background:**

Amnestic mild cognitive impairment (aMCI), considered as the prodromal stage of Alzheimer’s disease, is characterized by isolated memory impairment and cerebral gray matter volume (GMV) alterations. Previous structural MRI studies in aMCI have been mainly based on univariate statistics using voxel-based morphometry.

**Objective:**

We investigated structural network differences between aMCI patients and cognitively normal older adults by using source-based morphometry, a multivariate approach that considers the relationship between voxels of various parts of the brain.

**Methods:**

Ninety-one aMCI patients and 80 cognitively normal controls underwent structural MRI and neuropsychological assessment. Spatially independent components (ICs) that covaried between participants were estimated and a multivariate analysis of covariance was performed with ICs as dependent variables, diagnosis as independent variable, and age, sex, education level, and site as covariates.

**Results:**

aMCI patients exhibited reduced GMV in the precentral, temporo-cerebellar, frontal, and temporal network, and increased GMV in the left superior parietal network compared to controls (pFWER < 0.05, Holm-Bonferroni correction). Moreover, we found that diagnosis, more specifically aMCI, moderated the positive relationship between occipital network and Mini-Mental State Examination scores (pFWER < 0.05, Holm-Bonferroni correction).

**Conclusions:**

Our results showed GMV alterations in temporo-fronto-parieto-cerebellar networks in aMCI, extending previous results obtained with univariate approaches.

## INTRODUCTION

Mild cognitive impairment (MCI) is considered as an intermediate stage between normal aging and Alzheimer’s disease (AD), in which patients do not meet the criteria for dementia but they show objective cognitive impairment beyond that expected for their age and education while having normal capabilities to carry out everyday activities.^1–3^ Amnestic MCI (aMCI) is a subtype of MCI predominantly marked by impairment of episodic memory^4,5^ that has been linked to an increased risk of progression to AD. Annually, 10–15% of aMCI patients were reported to progress to AD and 80% of them convert into AD within six years.^2,6^ Therefore, a better understanding of brain changes in aMCI may contribute to enhance the treatment of early symptoms and to slowdown of disease progression.

Previous meta-analytic studies in aMCI have reported gray matter (GM) atrophy in subcortical regions such as the nucleus basalis of Meynert, amygdala, hippocampus and thalamus, as well as in a wide variety of cortical regions.^7,8^ The atrophy in frontal, temporal and parietal-occipital regions were reported in studies using various imaging modalities in aMCI.^9–14^ A recent study using three different methods to analyze the pattern of GM atrophy (two based on gray matter volume (GMV) and one on cortical thickness) in 27 aMCI and 58 controls revealed a pattern of temporo-fronto-parietal atrophy with source-based morphometry (SBM), anterior and posterior cingulate atrophy with voxel-based morphometry (VBM), and thinning of fronto-occipital areas using cortical surface-based analysis.^15^ Cortical thinning in aMCI has also been found in temporal, superior lateral parietal, prefrontal cortex and frontal lobes, as well in the precuneus.^16–18^ Remarkably, other studies have also reported cortical thickening in older individuals at risk for AD showing abnormal amyloid-β (Aβ) levels.^19–21^

The vast majority of the structural findings in MCI were obtained with VBM, a univariate statistical approach that is commonly used to assess group differences in local GM or white matter (WM) signal intensity using a voxel-wise comparison approach.^22^ As VBM focuses on regional differences, it may not unveil complex associations between distant brain regions that may parallel a similar level of atrophy. To counteract this drawback, here we have applied the multivariate SBM approach, which comprises voxels into separate networks that exhibit similar information and capture shared GMV features,^23,24^ to identify GM structural network alterations capable of distinguishing aMCI from cognitively normal older adults. To date, only one study has employed SBM to investigate GMV changes in a relatively small sample of 27 aMCI patients compared to healthy controls.^15^ A larger sample would allow for higher sensitivity in detecting subtle GMV alterations across the whole brain. We hypothesized that, relative to cognitively healthy elderly, aMCI patients would show GMV alterations in widespread networks encompassing frontal, temporal and parietal regions.

## MATERIALS AND METHODS

### Participants

One hundred and seventy-one participants, comprising 91 aMCI and 80 cognitively normal older adults (control group) were included in the analysis. Participants were recruited from two centers: 1) University of Medical Centre Groningen (UMCG), Groningen, The Netherlands (*n* = 51; 20 controls and 31 aMCI patients) and 2) Pablo de Olavide University, Seville, Spain (*n* = 120; 60 controls and 60 aMCI patients). A written informed consent was obtained from all participants and studies were approved by the Ethical Committee for Clinical Research of University Medical Center Groningen and the Junta de Andalucía, according to the principles outlined in the Declaration of Helsinki.

aMCI participants were primarily recruited from memory clinics. Diagnosis of aMCI was established by Petersen’s criteria,^3^ which requires presence of an isolated memory disorder with no impairment in other cognitive domains. The diagnosis was determined by a trained neuropsychologist and further confirmed by a neurologist. Cognitively normal older adults were recruited through advertisements and from senior citizen’s associations, health-screening programs, and hospital outpatient services. Inclusion criteria for the control group were - no subjective memory complaints (at UPO-Seville it was assessed with the Spanish version of the Memory Functioning Questionnaire),^25^ no objective memory complaints, as indicated by normal cognitive performance on neuropsychological assessment relative to appropriate reference values for age and education, a Clinical Dementia Rating scale global score of 0 (no dementia), and normal independent function. General exclusion criteria included MRI contra-indications, the anatomical abnormalities (e.g., brain tumor, cerebral infarction, hippocampal sclerosis, intracranial mass, large periventricular/deep WM lesions, and/or vascular malformations) found on the MRI scan, epilepsy, head trauma accompanied by a loss of consciousness, history of neurodevelopmental disease, mental retardation, alcohol abuse, hydrocephalus, and any current or history of psychiatric or neurological disorders, with the exception of depressive symptoms. All participants were not on any medication that might affect cognition at recruitment or during the study. At the UPO-Seville cohort, the absence of secondary causes of cognitive deficits (e.g., thyroid function, vitamin B12/folate) was confirmed with laboratory tests.

At UMCG, the education level was determined using the Dutch Verhage^26^ scores (ranging from 1 to 7) which classified into low (Verhage 1 and 2), middle (Verhage 3, 4, and 5), and high (Verhage 6 and 7). They correspond with US years of education as follows: Verhage 1:1 to 5 years; 2:6 years; 3:7 to 8 years; 4:7 to 9 years; 5:7–10 years; 6:7–16 years; and 7:17–20 years.^27^ Subsequently, we classified years of education from both centers into the following three categories: low (1–6 years), middle (7–10 years), and high (≥11 years of education).

### Behavioral and neuropsychological assessment

The 30-item Geriatric Depression Scale (GDS) was used to assess depressive symptoms, consisting of a self-report screening scale containing 30 “yes/no” questions with higher scores indicating more depressive symptoms.^28^ A set of neuropsychological assessments was administered, including the Mini-Mental State Examination (MMSE) as a measure of global cognitive function^29^ and the two forms of Trail Making Test (TMT part A and B) as measures of executive function.^30,31^

### Statistical analysis of demographic data

Statistical analyses for demographic, behavioral and neuropsychological data were performed using the IBM SPSS Statistics for Windows, Version 28.0 (IBM Corp, Armonk, NY, 2021). We first assessed the normality assumption of all the variables using the Shapiro-Wilk test. Group differences were analyzed using nonparametric Mann-Whitney U test for non-normally distributed data (i.e., age, MMSE, GDS-30, TMT A, and TMT B), and Chi-square tests for sex and education level. Level of significance for all tests was set at *p* < 0.05 (2-tailed).

### Neuroimaging methods

#### Magnetic resonance imaging acquisition

At the UMCG, MRI data were acquired using a 3.0-Tesla Philips Intera scanner (Philips Medical Systems, Best, The Netherlands) equipped with a 32-channel synergy SENSE head coil for excitation and signal collection. High-resolution three-dimensional T1-weighted images were acquired with the following acquisition parameters (repetition time [TR] = 9 ms; echo time [TE] = 3.6 ms; field of view [FOV] = 232×170×256 mm; voxel size = 1×1×1 mm; flip angle [FA] = 8°; acquisition time = 4.2 min). At the UPO-Seville cohort, MRI data were acquired using a 3.0-Tesla Philips Achieva scanner (Philips Medical Systems, Best, The Netherlands) equipped with an 8-channel head coil for excitation and signal collection. High-resolution three-dimensional T1-weighted images were acquired with the following acquisition parameters (repetition time [TR] = 11 ms; echo time [TE] = 4.5 ms; field of view [FOV] = 250×250×183 mm; 0.8×0.8×0.8 mm isotropic voxel resolution; flip angle [FA] = 8°; acquisition time = 9.1 min).

### Image preprocessing

The data were preprocessed and analyzed using Statistical Parametric Mapping (SPM12 v.7487; http://www.fil.ion.ucl.ac.uk/spm/) implemented in MATLAB 2015a (Mathworks Inc., Natick, MA, USA). First, the PAR/REC-files were converted to NIfTI, using an in-house script. Then the T1-images were reoriented manually to the Anterior Commissure-Posterior Commissure (AC-PC) plane and segmented into six different compartments (i.e., GM, WM, cerebrospinal fluid, bone, soft tissue, and air) with bias correction. The diffeomorphic anatomical registration through exponential lie algebra (DARTEL) tools was used to register the images.^32^ First, a template for the DARTEL procedures was created using the data from all participants using default parameter settings. The modulated GM images were spatially normalized to Montreal Neurological Institute (MNI) atlas space, resampled to 1.5×1.5×1.5 mm voxels, and smoothed using an 8 mm Full Width Half Maximum Gaussian kernel to increase signal to noise ratio.

### Source-based morphometry analysis

SBM is a multivariate approach used to identify naturally grouping patterns of GM volume variations among participants using independent component analysis (ICA). ICA captures and separates signals from sMRI images and identifies spatially independent components (ICs) that covary between participants.^23^

The SBM analysis was carried out with the Group ICA fMRI Toolbox (GIFT) software v4.0b (http://mialab.mrn.org/software/gift/). Nineteen ICs were automatically estimated by GIFT using the minimum description length method.^33^ ICA was performed using an Infomax algorithm that attempted to maximize the recognition of the independent components by exploiting signal intensities from the images.^34,35^ The component reliability was assessed with ICASSO (http://research.ics.aalto.fi/ica/icasso/) using 20 iterations. Reliability was quantified using a quality index (Iq) that ranges between 0 and 1.^36^ All 19 components extracted from the GM images showed an Iq > 0.95, indicating a highly stable ICA decomposition, and therefore they were subsequently included in the rest of the analysis.

Each GM volume was converted into a one-dimensional vector by SBM. In this study, we obtained a matrix comprised of 171 rows which represented 171 participants (the first 80 rows represent controls, and the following 91 rows aMCI) and each column indicated a voxel. This matrix was decomposed into two matrices by ICA, resulting in a “mixing matrix” and “source matrix”. The mixing matrix comprised one subject per row and one IC per column. The values of this mixing matrix are called “loading coefficients” which demonstrates how representative each subject is on the specific component and were subsequently used for statistical analysis. The latter matrix is the source matrix, which shows the relation between the ICs and the voxels. To visualize the GMV component, the source matrix was converted into a 3D image, scaled to unit standard deviation (Z maps) and thresholded at |Z | = 3, thus showing only the voxels that strongly contributed to these components. Maps of components showing significant differences between aMCI and controls were then overlaid onto MNI normalized anatomical atlas. The anatomic region was defined according to automated anatomical labeling atlas^37^ based on the transformed locations of the largest clusters in the component maps and were visually confirmed using MRIcroGL (https://www.nitrc.org/projects/mricrogl/).

A greater loading coefficient indicates that the group’s corresponding spatial pattern has stronger weight than the other group. The sign of the loading coefficients of a component alone does not directly provide the direction of change in absolute GMV in a region. For example, if the group mean loadings show aMCI > controls and the spatial component is predominantly comprised of negative voxels, it can be inferred that GM volume is lower in aMCI than in controls. The interpretation of differences in the loading coefficient should therefore be carefully done as it depends upon the spatial distribution of the component.^15,38^

### Statistical analysis

We applied the Yeo-Johnson transformation to the ICs to improve normality and alleviate heteroscedasticity.^39^ One subject was removed from analysis since it was detected as an outlier via the Mahalanobis distance outlier detection.

First, a Multivariate Analysis of Covariance (MANCOVA) was performed to assess the main effect of diagnosis on the 19 ICs. The multiple linear regression model was created using the *fitlm* function in MATLAB, with 19 ICs as dependent variables, diagnosis as independent variable, and age, sex, education level and site as covariates. We also analyzed the effect of site on the 19 ICs while controlling for age, diagnosis, sex, and education level. The analysis was performed using in-house developed scripts in MATLAB.

Second, we performed receiver operating characteristic (ROC) curve using logistic regression analyses to investigate whether significant ICs identified by the MANCOVA were able to distinguish between aMCI and controls, adjusting by the same covariates. We conducted a cross-validation procedure with 1000 permutations and calculated the Youden’s index, area under the curve (AUC), and the overall accuracy to assess the performance of the logistic regression model. Each score ranged from 0 to 1, with higher values indicating better performance. In addition, we performed relative weight analysis to determine the magnitude of each predictor and their contribution to the model.

Third, we evaluated 1) whether ICs were associated with MMSE, GDS-30, TMT A, and TMT B, regardless of diagnosis, and 2) whether the interaction between diagnosis and ICs accounted for the variability in MMSE, GDS-30, TMT A, and TMT B. All statistical models were adjusted for age, sex, education level, and site. Three cases who had no data for the TMT test and one outlier were removed from further analyses.

For all analyses, we report *p*-values corrected for multiple comparisons using the Holm-Bonferroni (*pFWER* < 0.05, 2-tailed) and the False Discovery Rate (FDR) methods. In addition, we calculated the Bayes factor (BF_10_) to assess the evidence in favor of the alternative hypothesis relative to the null hypothesis. We used the classification by Lee & Wagenmakers, where larger BF_10_ value indicates stronger evidence in favor of the alternative hypothesis.^40^ The standardized effect size (Cohen’s g) was obtained to estimate the magnitude of difference between groups and categorized as small effect (0.2–0.5), moderate effect (0.5–0.8), and large effect (>0.8).^41^ We then estimated the accuracy of the effect size by calculating the bias-corrected and accelerated (BCa) bootstrap 95% confidence intervals (CI_95 %_).

## RESULTS

### Participants’ characteristics

Table 1 shows demographic, behavioral, and neuropsychological data of the study sample. There were no significant differences between groups on age, sex, and education level. As expected, the aMCI group showed significantly lower global cognition scores as measured by the MMSE and executive function as measured by TMT A and B. The aMCI group also showed higher GDS-30 scores relative to controls. Using the GDS-30’s cut-off score of 11,^28^ 80 participants with aMCI and 79 controls did not meet the criteria for depression (scored 0–10), 9 aMCI and 1 control exhibited mild depressive symptoms (scored 11–20), and 2 aMCI patients were considered to have moderate to severe depressive symptoms (scored above 20).

**Table 1:** Demographic characteristics

	aMCI (*n* = 91)		Control (*n* = 80)		Group comparison (*p*)
	Mean (SD)	Range	Mean (SD)	Range
Age	68.9 (5.9)	51–83	68.1 (3.9)	61–79	U = 3289.5 (0.28)
Sex Female, *n* (%)	35 (38.5)		32 (40)		*χ*^2^ = 0.04 (0.84)
Education category, *n* (%)					*χ*^2^ = 1.27 (0.53)
Low	64 (70.3)		50 (62.5)
Middle	11 (12.1)		11 (13.7)
High	16 (17.6)		19 (23.8)
MMSE	27.6 (2.2)	22–30	29 (1.1)	26–30	U = 2115 (<0.001)^*^
GDS-30	3.5 (5.4)	0–25	1.2 (2.1)	0–12	U = 2716.5 (0.003)^*^
TMT A	46.5 (22.5)	20–147	38.2 (14.3)	15–94	U = 2660.5 (0.003)^*^
TMT B	134.3 (72.4)	40–539	103.7 (54.6)	39–360	U = 2450 (<0.001)^*^

Group comparisons were performed with Mann-Whitney *U*-test for age, MMSE, GDS-30, TMT A, TMT B, and Pearson’s chi-square tests for sex and educational level. aMCI, amnestic Mild Cognitive Impairment; MMSE, Mini-Mental State Examination; GDS, Geriatric Depression Scale; TMT A and B, Trail Making Test (forms A and B) in seconds. ^*^p < 0.05.

### Group differences in cerebral gray matter structural networks

**Fig. 1:**
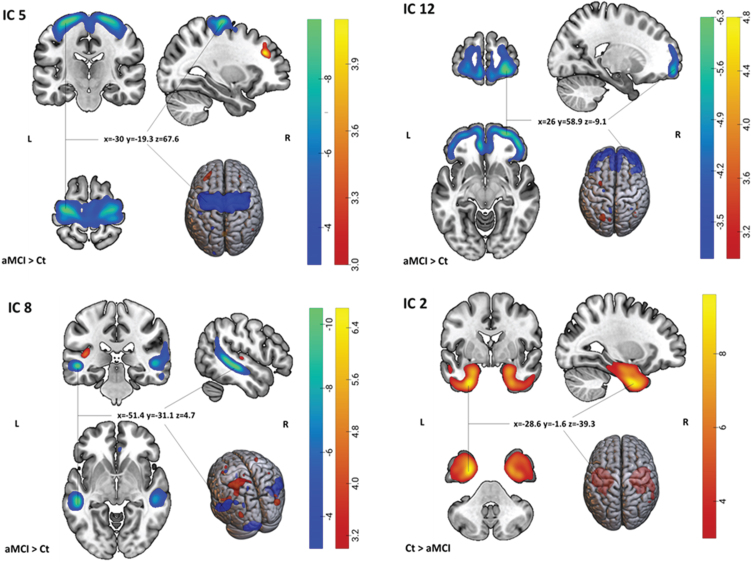
Spatial maps of the 4 ICs showing significant group differences (aMCI versus Ct). For IC 5, 8, and 12: Light-dark blue colored regions show decreased GM in aMCI relative to Ct. Red-yellow-colored regions show increased gray matter in aMCI relative to Ct. For IC 2: Red-yellow-colored regions show increased gray matter in Ct relative to aMCI (or vice versa, decreased gray matter in aMCI relative to Ct). x,y,z, MNI coordinates of cluster maximum intensity. The color bar indicates the color mapping for the normalized component weights (Z-scores, thresholded at | 3 |). aMCI, amnestic mild cognitive impairment; Ct, controls; IC, Independent Component.

Nineteen independent components (GM structural networks) were estimated with ICA. There were no obvious artifacts observed on visual inspection. As shown in Table 2 and Fig. 1, after adjusting for age, sex, education level, and site, MANCOVA revealed significant main effects of diagnosis on four components (ICs): IC 2, 5, 8, and 12 which survived correction for multiple comparisons.

**Table 2:** SBM components showing significant differences between aMCI and Controls ordered by the decreasing BF10 value

	*pFWER*	*pFDR*	R^2^	F(1,164)	Effect size (G)	CI_95 %_	BF_10_
IC 5	<0.0001	<0.0001	0.53	0.06	–0.99^L^	–1.42 ––0.52	10.39^S^
IC 8	<0.005	<0.005	0.21	18.49	–0.64^M^	–0.96 – –0.30	5.02^M^
IC 12	0.02	0.01	0.22	24.38	–0.59^M^	–0.91 – –0.24	3.43^M^
IC 2	0.03	0.01	0.37	45.10	0.61^M^	0.25–0.97	3.14^M^

Statistical significance was set to pFWER < 0.05, Holm-Bonferroni correction. Effect size Cohen’s G (^L^large, ^M^moderate); BF_10_: magnitude of the evidence in favor of the alternative hypothesis (^S^strong, ^M^moderate).

**Fig. 2:**
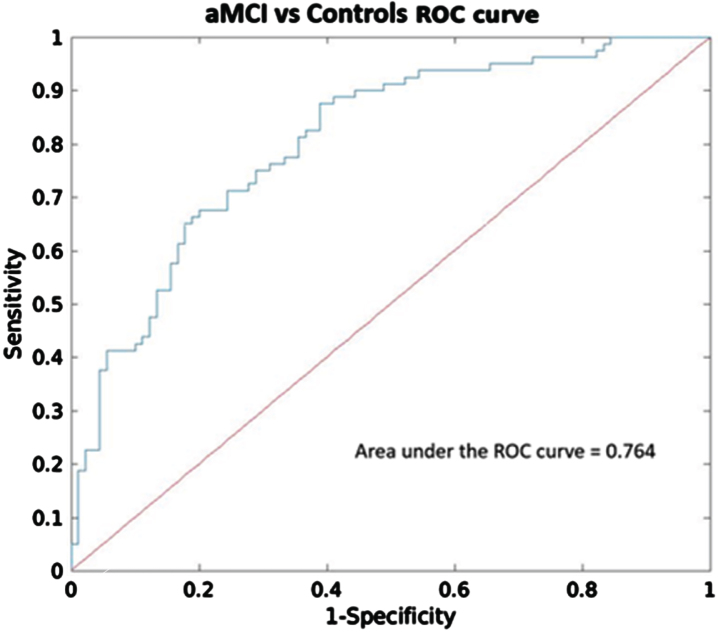
Receiver operating characteristic (ROC) analysis of components that showed significant group differences (IC 2, 5, 8, and 12). ROC curve and the corresponding area under the curve (AUC) to differentiate between aMCI and Controls.

After performing the logistic regression analysis with these four ICs, two components, IC 5 (*p* < 0.0001) and IC 8 (*p* < 0.05), exhibited significant diagnostic accuracy in distinguishing aMCI and controls, with Youden’s index of 0.56, overall accuracy of 79.4%, and AUC of 0.76 (CI_95 %_ = 0.70–0.82) (Fig. 2). Additionally, the relative weight analysis showed that IC 5 had the highest relative weight (0.13) and rescaled relative weight (0.46) with CI_95 %_ = 0.04–0.30 among the other significant components.

To evaluate the predictive capacity of these IC networks (IC 5 and IC 8) compared to the hippocampal volume, we employed additional logistic regression analyses. First, we built a model using overall hippocampal volume (i.e., sum of bilateral volumes relative to total intracranial volume, TIV) alone with age, site, sex, and education level as covariates. As expected, hippocampal volume (*p* < 0.0001) exhibited significant diagnostic accuracy in distinguishing aMCI and controls, with Youden’s index of 0.53, overall accuracy of 78%, and AUC of 0.72 (CI_95 %_ = 0.65–0.78). Second, we developed an additional model by incorporating IC 5, IC 8 and hippocampal volume as regressors of interest together with the nuisance variables. IC 5 (*p* < 0.0001), IC 8 (*p* < 0.05) and hippocampal volume (*p* < 0.005) also showed good discrimination ability with Youden’s index of 0.60, overall accuracy of 81%, and AUC of 0.78 (CI_95 %_ = 0.72–0.83). In this model, IC 5 had a higher relative weight (0.11) and rescaled relative weight (0.34) with CI_95 %_ = 0.05–0.43 compared to the hippocampal volume (relative weight = 0.09, rescaled relative weight = 0.29, CI_95 %_ = 0.04–0.21). Lastly, we compared the model including IC 5 and IC 8, and another model including hippocampal volume and the covariates. Using Wilcoxon-Test, the model incorporating IC 5 and IC 8 exhibited significantly better accuracy (*p* < 0.0001) compared to the model comprising hippocampal volume alone. However, there was no significant difference in AUC between the two models (*p* = 0.18).

The largest significant difference between aMCI and controls was shown by IC 5, which had greater loading weights (i.e., a combination of volume and covariation between the volumes in each voxel within the component) in the aMCI group compared to controls (a loading directionality of aMCI > Ct). This component consisted mainly of negative voxels in the precentral gyrus, indicating lower GMV in aMCI compared to controls. IC 8 included areas with both negative and positive voxels, and it had greater loading weight in aMCI than in controls. The negative regions were primarily located in bilateral middle temporal gyrus and cerebellum, while the positive regions mostly included the left superior parietal lobule. IC 12 included negative voxels in bilateral middle frontal gyrus. The loading directionality for this component was aMCI > Ct. IC 2 showed greater loading weights in controls compared to aMCI (Ct > aMCI) and affected areas containing positive voxels mostly in bilateral fusiform gyrus and parahippocampal gyrus (see Supplementary Table 1 and Supplementary Figures 1–4 for all regions comprising the significant components).

### Relationship between site and ICs and the interaction between site and diagnosis with ICs

MANCOVA revealed significant main effects of site on five components, i.e., IC 5, 7, 15, 16, and 19 (See Supplementary Table 4). Among these, IC 5 is particularly noteworthy due to its superior discrimination ability between controls and aMCI. However, we found no significant interaction effects between site and diagnosis on any of the ICs.

### Relationship between ICs and MMSE, GDS-30, TMT A, and TMT B

**Fig. 3:**
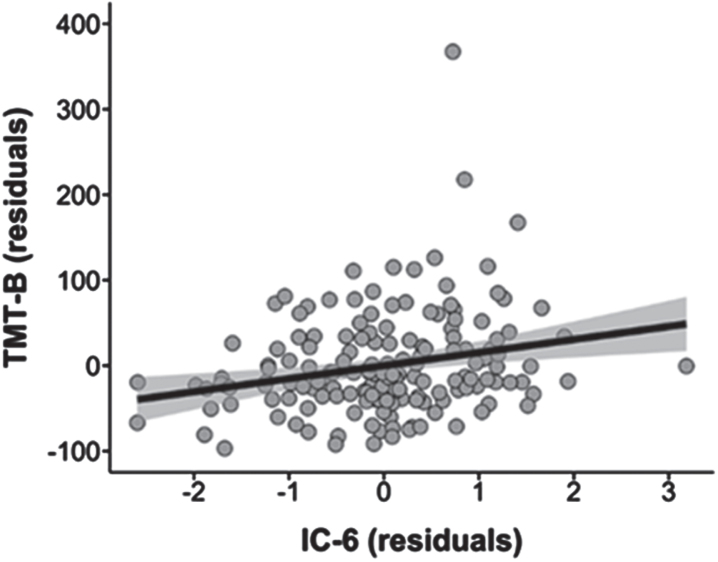
Association between IC 6 and TMT B scores in the whole sample (*pFDR* < 0.05, *pFWER* Holm-Bonferroni = 0.26). IC, Independent Component; TMT B, Trail Making Test Part B.

We found a positive relationship between IC 6 and TMT B regardless of the diagnosis (*t* = 2.29, *pFDR* < 0.05) but did not survive the corrected significance threshold (pFWER Holm-Bonferroni = 0.26, BF_10_ = 4.98) (Fig. 3, Supplementary Table 2). There was no significant relationship between other ICs and MMSE, GDS-30, or TMT A.

### Relationship of the interaction between ICs and diagnosis with MMSE, GDS-30, TMT A, and TMT B

**Fig. 4:**
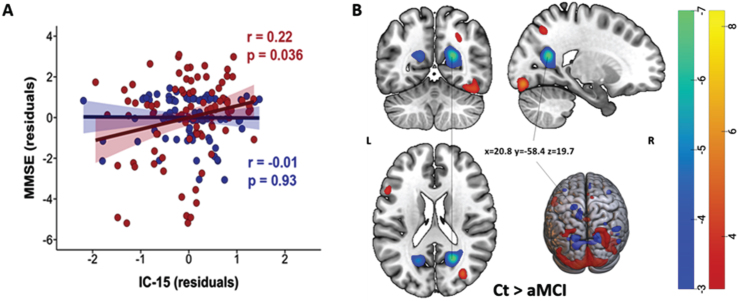
A) Significant positive correlations between IC 15 and diagnosis’ interaction and MMSE in the aMCI group (red, *pFDR* = 0.036, *pFWER* Holm-Bonferroni = 0.02). Negative correlations in the Ct group were not significant and are displayed in blue. B) Spatial map of IC 15. Light-dark blue colored regions show decreased gray matter in Ct relative to aMCI (or vice versa, increased gray matter in aMCI relative to Ct). Red-yellow colored regions showed decreased gray matter in aMCI relative to Ct. (Z-scores, thresholded at | 3 |). x,y,z, MNI coordinates of cluster maximum intensity. The color bar indicates the color mapping for the normalized component weights (Z-scores, thresholded at | 3 |). aMCI, amnestic mild cognitive impairment; Ct, controls; IC, Independent Component; MMSE, Mini-Mental State Examination.

On assessing the interaction between ICs and diagnosis, a significant interaction effect was observed between IC 15 and diagnosis on MMSE scores. As displayed in Fig. 4A, the loading in IC 15 showed significant positive correlations with the MMSE scores only in the aMCI group (red) (*r* = 0.22, *pFDR* = 0.04, *pFWER* Holm-Bonferroni = 0.02, BF_10_ = 41.9). Note that the spatial distribution of IC 15 showed loading directionality of aMCI < Ct and included both positive (red) and negative (blue) voxels (Fig. 4B). Thus, our result indicated that among aMCI patients, higher MMSE scores were associated with alterations mainly in the right calcarine region (blue-green), while lower MMSE scores were associated with alterations mainly in the left calcarine, right occipital superior gyrus and fusiform gyrus (red-yellow). The association of ICs with GDS-30, TMT A, or TMT B did not differ between groups.

## DISCUSSION

The present study utilized SBM, a data-driven and multivariate method, to investigate differences in GM structural networks between aMCI and cognitively normal older individuals. Particularly, aMCI patients exhibited reduced GMV mainly in the precentral network (IC 5), temporo-cerebellar network (IC 8), frontal network (IC 12), and temporal network (IC 2), and increased GMV in the left superior parietal network (IC 8) compared to controls. While the effect of site was significant on IC 5, the group differences observed in IC 2, 5, 8, and 12 were not modulated by site. Moreover, we found a positive relationship between diagnosis and alterations in the occipital network (IC 15) with MMSE specifically in aMCI group, indicating that the effect of alterations in the occipital network on MMSE scores was more pronounced among aMCI patients compared to cognitively healthy elderly.

The atrophy detected in the precentral network (IC 5) in aMCI patients is in line with previous studies that have reported a loss of GM in the same regions in both MCI and AD patients.^12,16,42^ This network mainly involves the precentral gyrus, also known as the primary motor cortex, and extends to the postcentral gyrus (primary somatosensory cortex). The primary motor cortex plays a significant role in initiating and regulating complex voluntary movements. Moreover, decreased GMV in parts of the primary somatosensory cortex may affect the process of somatosensory feedback and the integration of sensory and motor signals necessary for voluntary movement^43^ to the brainstem and spinal cord through motor pathways such as corticospinal and corticobulbar tracts.^44^ Thus, the decreased GMV found in the precentral network and connected area could be associated with a deficit in motor control and coordination and impaired sensorimotor function in aMCI patients. A number of studies have also emphasized the role of motor cortex and somatosensory cortex in emotional regulation and complex cognitive functions such as spatial navigation^45–48^ which are affected in AD. This further supports the involvement of the precentral gyrus in aMCI. Furthermore, changes in sensory and motor function may increase the risk of AD and may occur earlier than cognitive symptoms in AD.^49^

Our findings of decreased GMV in the temporo-cerebellar network (IC 8) in aMCI were mainly located in bilateral middle temporal gyrus and posterior cerebellum. The GMV atrophy found in the temporal lobe is largely in line with the result derived from a recent meta-analysis of 45 studies^8^ and with previous neuroimaging reports in both MCI and AD.^7,18,50–52^ The pattern of cerebellum and lateral temporal network atrophy in this study was not found in a previous study using SBM, whose results were limited to the temporo-fronto-parietal network.^15^ However, there is evidence of an increased cerebellar atrophy with disease progression from normal aging to MCI and further to AD.^53^ This pattern of cerebellar GMV atrophy has been reported to occur first in the vermis in the anterior cerebellum in aMCI extending to the hemispheric part of the posterior cerebellum and Crus I in AD.^54^ Although the cerebellum is considered essential for sensorimotor and posture control,^55^ recent studies have suggested its role in non-motor function, including cognition, emotion, behavior, and autonomic function.^53,54,56,57^ Here, we found that reduced cerebellar GMV was mainly located in lobules 8 and 9 (see Supplementary Table 1) suggesting that the atrophy in aMCI was mostly located in the posterior area. Posterior cerebellar atrophy has been associated with poorer cognitive functioning in AD patients compared to healthy controls^58^ and with executive functioning in community-dwelling older persons.^59^ However, cerebellar volume has also been negatively associated with cognition in MCI.^53^ The role of the cerebellum in cognitive functioning might be explained by the reciprocal connections of the cerebellum with different brain regions that are involved in cognition and behavior, such as prefrontal, temporal, posterior parietal, and limbic cortices.^53,56,57^

Interestingly, we also found the co-occurrence of increased GMV in the left superior parietal lobe and decreased GMV in the temporo-cerebellar network. Increased cortical volume/thickness has been related to AD pathology that appears long before the onset of clinical AD symptoms.^19–21,60^ A previous study reported that cognitively normal controls with high Aβ deposition had a larger temporal lobe (including hippocampal/parahippocampal gyrus area) than healthy controls with low Aβ deposition.^20^ A study in people at high risk for AD also showed increased GM in bilateral lateral parietal lobe in the group with positive Aβ (Aβ+) compared to the Aβ– group, although these differences did not survive multiple comparison corrections.^19^ Increased cortical thickness in the middle temporal,^21^ temporoparietal, and precuneus^60^ has been reported in Aβ+ cognitively healthy subjects. This anomalous increase in GM could result from a reactive neuronal hypertrophy and/or amyloid-driven inflammation in the early stage of the disease;^60^ however, it could also signal brain reserve or a compensatory process in response to toxic effects of Aβ or diffuse plaques.^20^

As expected, decreased GMV was also detected in the prefrontal cortex (IC 12) and medial temporal network (IC 2). The prefrontal cortex supports executive functioning, including cognitive control processes for memory function (e.g., selection, engagement, monitoring, and inhibition), while the medial temporal lobe is vital for encoding, storage, and retrieval of long-term memories.^61^ Patterns of GM atrophy found in the medial temporal network are consistent with the notion that medial temporal regions are the first region to be affected before AD pathology spreads to posterior cingulate cortex, temporo-parietal association cortex, prefrontal and the orbital frontal cortex in MCI and AD patients.^62–64^ The medial temporal lobe, including the hippocampus, amygdala, and parahippocampal regions, plays an important role for episodic and spatial memory as it involves distinct processes as encoding (i.e., transforming the perceived information into a memory trace), consolidation (i.e., stabilizing memory traces process), and memory retrieval/recall (i.e., the process by which memory traces are reactivated to access information previously encoded and stored in the brain).^65^ Decreased cortical thickness in the fusiform gyrus of both hemispheres has been reported from cognitively normal elderly to single- and multiple-domain aMCI to AD patients finally.^66^ We did not find a significant association between the executive functioning tests (TMT-A or B) and spatial patterns of GMV. However, a significant interaction effect was found between diagnosis and the occipital network with MMSE in the aMCI group (see Supplementary Table 3). Occipital region is crucial for processing visual information for spatial orientation,^67^ which is highly affected in AD.^68^ Visuospatial function impairment has been reported to occur in early AD.^69^ Another study found a significant positive correlation between atrophy in left superior and middle temporal gyrus and poorer global cognition in aMCI.^16^ However, this finding needs to be confirmed in independent studies.

Our results showing GMV alterations in aMCI are more widespread than in the previous SBM study.^15^ A previous longitudinal study in aMCI patients found widespread patterns of cortical thinning mainly in the temporal, superior lateral parietal and some regions of the frontal cortices in aMCI.^16^ Another study showed cortical thinning in temporal and insular regions in early aMCI, and in more widespread regions in late-stage aMCI, including the bilateral dorsolateral prefrontal, anterior and medial temporal cortex, temporo-parietal association cortices, and the precuneus.^17^

Although our findings indicate that both hippocampal and IC networks contribute to distinguishing aMCI and controls, IC 5 appears to play a more prominent role in predictive modeling. The higher accuracy attained by the model incorporating IC 5 and IC 8 highlights the potential of the ICs networks derived by SBM approach as complementary predictors alongside hippocampal volume in aMCI diagnosis.

### Strengths and limitations

The use of SBM to determine GMV loss is a strength of our study as it reveals similar covariance patterns and reduces the problem of multiple comparisons. This study also included a large sample size from two independent cohorts. However, some limitations that could affect the results and their interpretation should also be considered. This is a cross-sectional study where changes in GMV over time cannot be determined. Moreover, the aMCI group may be heterogeneous due to their recruitment in different memory clinics and the lack of biomarker data (i.e., PET imaging, cerebrospinal fluid, or blood) confirming the presence of AD pathology. As such, several different etiologies, presentations and outcomes (i.e., single- versus multiple-domain; early- versus late-stage; stable- versus progressive-aMCI) are possible, and they may be responsible for the widespread atrophy patterns found in the present study. The participants did not undergo a comprehensive neuropsychological test-battery, which would enable us to further assess whether the increase in GMV is reflecting a reactive response or a compensatory process. Further longitudinal studies are needed to better explain the observed changes. Lastly, this study is also limited by the absence of a validation cohort to confirm the generalizability of the findings. Future research with a validation cohort is necessary to determine the diagnostic value in a broader clinical setting.

### Conclusions

In conclusion, our results showed GMV alterations in temporo-fronto-parieto-cerebellar networks in aMCI confirming previous findings in aMCI and AD, but these changes are more extensive than reported previously. These results suggest that SBM may be a more sensitive approach to monitor brain changes in prodromal AD, which may be employed as a target for early interventions before AD diagnosis.

## AUTHOR CONTRIBUTIONS

Tania M. Setiadi (Conceptualization; Formal analysis; Methodology; Visualization; Writing – original draft); Jan-Bernard C. Marsman (Formal analysis; Methodology; Software; Writing – review & editing); Sander Martens (Methodology; Supervision); Shankar Tumati (Conceptualization; Funding acquisition; Investigation; Writing – review & editing); Esther M. Opmeer (Investigation; Supervision; Writing – review & editing); Fransje E. Reesink (Investigation; Writing – review & editing); Peter P. De Deyn (Conceptualization; Investigation; Writing – review & editing); Mercedes Atienza (Formal analysis; Funding acquisition; Methodology; Software; Writing – review & editing); André Aleman (Conceptualization; Funding acquisition; Supervision; Writing – review & editing); Jose L. Cantero (Conceptualization; Funding acquisition; Writing – review & editing).

## Supplementary Material

Supplementary Material

## Data Availability

The data are not publicly available due to privacy or ethical restrictions.
