# Supplementary material for: Alterations in Gray Matter Structural Networks in Amnestic Mild Cognitive Impairment: A Source-Based Morphometry Study
Source: J Alzheimers Dis. 2024 Aug 27;101(1):61–73. doi: 10.3233/JAD-231196 (PMC11380280; doi:10.3233/JAD-231196)
Supplement: Supplementary Material [file jad-101-jad231196-s001.pdf]

Supplementary Material

Alterations in Gray Matter Structural Networks in Amnestic Mild Cognitive Impairment: A Source-Based Morphometry Study

Supplementary Table 1. List of brain regions comprising SBM components that shows significant group (aMCI/Ct) effect

Component	Peak brain region (AAL)[a]	Z max	Volume[b]	Peak coordinates[c]			Cluster regional extent[d]
				x	y	z	
IC 5	Negative weighted						
	Precentral_L	-9.6	49479*	-30.0	-19.3	67.6	Precentral_L&R, Paracentral_Lobule_L&R, Postcentral_L&R, Supp_Motor_Area_L&R, Frontal_Sup_R, Precuneus_L, Frontal_Mid_R
	Parietal_Sup_L	-3.6	174	-21.2	-65.8	44.8	Parietal_Sup_L, Occipital_Sup_L, Parietal_Inf_L
	Temporal_Sup_L	-3.5	161	-44.8	-27.4	8.6	Temporal_Sup_L, Heschl_L
	Occipital_Mid_L	-3.2	121	-18.2	-101.9	1.2	Occipital_Mid_L, Calcarine_L
	Positive weighted						
	Frontal_Mid_L	4.1	1649	-30.0	34.5	28.5	Frontal_Mid_L, Frontal_Sup_L, Frontal_Inf_Tri_L
	Frontal_Mid_R	3.2	57	29.0	36.0	28.5	Frontal_Mid_R
	Temporal_Inf_R	3.2	68	52.6	-51.0	-20.9	Temporal_Inf_R
IC 8	Negative weighted						
	Temporal_Mid_L	-10.5	11909*	-51.4	-31.1	-4.7	Temporal_Mid_L, Angular_L, Occipital_Mid_L, Temporal_Sup_L, SupraMarginal_L
	Temporal_Mid_R	-8.9	24832*	51.1	-34.8	1.2	Temporal_Mid_R, Temporal_Sup_R, Temporal_Inf_R, Angular_R, SupraMarginal_R,Parietal_Inf_R
	*	-6.1	5948*	0.2	-56.9	-51.1	Cerebelum_9_L, Cerebelum_9_R, Cerebelum_8_L, Cerebelum_8_R, Vermis_9
	Precentral_L	-3.6	127	-37.4	-9.0	61.7	Precentral_L
	Occipital_Mid_L	-3.5	196	-28.6	-73.9	25.6	Occipital_Mid_L, Occipital_Sup_L
	Cingulum_Ant_R	-3.3	142	4.6	37.5	-3.2	Cingulum_Ant_R, Frontal_Med_Orb_R
	Cingulum_Mid_L	-3.2	131	-6.4	-43.7	38.1	Cingulum_Mid_L, Precuneus_L
	Positive weighted						
	Parietal_Sup_L	6.7	6034*	-34.5	-51.0	58.8	Parietal_Sup_L, Parietal_Inf_L, Postcentral_L, Precuneus_L, Angular_L
	Occipital_Mid_R	5.6	887	40.8	-74.6	10.8	Occipital_Mid_R, Temporal_Mid_R
	Occipital_Mid_L	5.0	526	-35.9	-79.8	10.1	Occipital_Mid_L
	Temporal_Sup_L	4.6	1176	-40.4	-32.6	11.6	Temporal_Sup_L, Rolandic_Oper_L, Heschl_L
	Frontal_Mid_R	3.8	272	31.2	2.8	57.3	Frontal_Mid_R, Frontal_Sup_R
	Precentral_L	3.7	126	-37.4	5.8	46.2	Precentral_L
	Temporal_Mid_R	3.7	197	46.7	-55.5	8.6	Temporal_Mid_R
	Frontal_Mid_L	3.4	137	-31.5	5.0	58.8	Frontal_Mid_L, Precentral_L
	Parietal_Sup_R	3.2	48	37.1	-45.1	60.2	Parietal_Sup_R
	Precuneus_L	3.2	38	-15.3	-43.7	73.5	Precuneus_L, Parietal_Sup_L, Postcentral_L
IC 12	Negative weighted						
	Frontal_Mid_Orb_R	-6.3	58628*	26.0	58.9	-9.1	Frontal_Mid_L&R, Frontal_Mid_Orb_L&R, Frontal_Inf_Orb_L&R, Frontal_Med_Orb_L&R, Rectus_L, Cingulum_Ant_L, Frontal_Sup_L&R
	Precentral_L	-3.6	87	-22.7	-14.9	72.0	Precentral_L
	Cingulum_Post_L	-3.5	333	-7.9	-52.5	34.4	Precuneus_L, Cingulum_Post_L
	Angular_R	-3.3	51	34.9	-49.6	40.3	Parietal_Inf_R, Angular_R
	Positive weighted						
	Occipital_Sup_L	4.8	1576	-24.1	-68.0	35.9	Occipital_Sup_L, Occipital_Mid_L, Parietal_Sup_L, Cuneus_L, Parietal_Inf_L

	Precentral_L	3.7	352	-30.0	-1.6	55.1	Frontal_Mid_L, Precentral_L
	Temporal_Mid_R	3.3	65	43.7	-56.9	18.9	Temporal_Mid_R
	Cuneus_L	3.3	230	-7.9	-73.9	24.1	Cuneus_L, Calcarine_L
	Temporal_Mid_R	3.2	33	45.2	-64.3	2.7	Temporal_Mid_R
IC 2	Positive weighted						
	Fusiform_L	9.6	29084*	-28.6	-1.6	-39.3	Fusiform_L, Temporal_Inf_L, ParaHippocampal_L, Temporal_Pole_Mid_L, Hippocampus_L, Temporal_Mid_L, Temporal_Pole_Sup_L, Amygdala_L
	ParaHippocampal_R	7.6	30983*	23.8	-4.6	-23.8	Temporal_Inf_R, Fusiform_R, Temporal_Pole_Mid_R, ParaHippocampal_R, Hippocampus_R, Temporal_Pole_Sup_R, Amygdala_R, Temporal_Mid_R
	Temporal_Mid_R	3.3	115	54.0	-52.5	-1.0	Temporal_Mid_R, Temporal_Inf_R

[a]Anatomical region where the peak voxel is located using automated anatomical labeling (AAL); [b]The volume in each area is number of voxels multiply by volume in each voxel (provided in cubic millimeters:mm^3); [c]Peak stereotaxic coordinates are reported in Montreal Neurological Institute (MNI) space; [d]Anatomical regions associated with the cluster; Left (L) and Right (R) cortical hemisphere; *number of voxels>1000.

Supplementary Table 2. SBM component showing association with TMT B

Component	Peak brain region (AAL)[a]	Z max	Volume[b]	Peak coordinates[c]			Cluster regional extent[d]
				x	y	z	
IC 6	Negative weighted						
	Putamen_R	-10.1	19977*	26.7	6.5	-0.2	Putamen_R, Caudate_R, Insula_R, Pallidum_R, Amygdala_R, Olfactory_R
	Putamen_L	-9.4	18364*	-25.6	5.0	-0.2	Putamen_L, Insula_L, Caudate_L, Pallidum_L, Olfactory_L, Amygdala_L, Temporal_Sup_L
	Thalamus_L	-6.2	4458*	-10.9	-25.2	5.7	Thalamus_R, Thalamus_L
	Positive weighted						
	Precuneus_R	3.6	169	10.5	-49.6	34.4	Precuneus_R, Cingulum_Mid_R, Cingulum_Post_R

[a]Anatomical region where the peak voxel is located using automated anatomical labeling (AAL); [b]The volume in each area is number of voxels multiply by volume in each voxel (provided in cubic millimeters:mm^3); [c]Peak stereotaxic coordinates are reported in Montreal Neurological Institute (MNI) space; [d]Anatomical regions associated with the cluster; Left (L) and Right (R) cortical hemisphere; *number of voxels>1000.

Supplementary Table 3. SBM component showing significant interaction's effect associated with MMSE

Component	Peak brain region (AAL)[a]	Z max	Volume[b]	Peak coordinates[c]			Cluster regional extent[d]
				x	y	z	
IC 15	Negative weighted						
	Calcarine_R	-7.0	4758*	20.8	-58.4	19.7	Calcarine_R, Precuneus_R, Cuneus_R
	Angular_R	-5.1	1111	48.1	-52.5	31.5	Angular_R,Temporal_Sup_R
	Cuneus_L	-5.0	1581	-18.2	-61.4	19.7	Cuneus_L, Calcarine_L, Precuneus_L, Occipital_Sup_L
	Cingulum_Mid_L	-4.4	1646	-13.1	-31.1	43.3	Cingulum_Mid_L, Precuneus_L, Paracentral_Lobule_L
	Postcentral_L	-4.4	653	-19.7	-28.2	61.7	Precentral_L, Postcentral_L, Paracentral_Lobule_L
	Frontal_Inf_Tri_R	-4.4	450	39.3	40.4	8.6	Frontal_Mid_R, Frontal_Inf_Tri_R
	Temporal_Inf_R	-4.2	305	54.0	-50.3	-6.1	Temporal_Inf_R, Temporal_Mid_R
	Cerebelum_4_5_L	-4.1	3122	-14.6	-43.7	-19.4	Cerebelum_4_5_L, Cerebelum_4_5_R, Vermis_4_5, Cerebelum_6_L, Cerebelum_3_R, Vermis_3, Cerebelum_3_L
	Fusiform_L	-3.7	217	-30.0	-43.7	-7.6	Fusiform_L, ParaHippocampal_L, Lingual_L
	Angular_R	-3.6	116	31.2	-49.6	43.3	Parietal_Inf_R, Angular_R
	Frontal_Mid_L	-3.5	193	-34.5	30.1	31.5	Frontal_Mid_L, Frontal_Inf_Tri_L
	Cingulum_Mid_R	-3.5	288	7.6	27.1	41.8	Frontal_Sup_Medial_R, Cingulum_Mid_R, Cingulum_Ant_R
	Fusiform_R	-3.2	46	31.9	-41.4	-9.1	Fusiform_R, ParaHippocampal_R, Lingual_R
	Positive weighted						
	Calcarine_L	8.3	16702*	3.9	-87.2	-9.1	Lingual_R, Calcarine_L, Cerebelum_Crus1_L, Lingual_L, Occipital_Inf_L, Calcarine_R, Fusiform_L, Cerebelum_Crus1_R, Occipital_Inf_R, Cerebelum_6_R, Occipital_Mid_L, Cerebelum_Crus2_L, Fusiform_R
	Occipital_Sup_R	6.5	6520*	28.2	-64.3	37.4	Occipital_Mid_R, Occipital_Sup_R, Angular_R, Parietal_Sup_R, Precuneus_R
	Fusiform_R	5.0	4568*	42.2	-54.0	-17.9	Fusiform_R, Temporal_Inf_R, Occipital_Inf_R, Cerebelum_6_R, Cerebelum_Crus1_R
	Frontal_Inf_Oper_L	4.7	1212	-54.4	15.3	13.0	Frontal_Inf_Oper_L, Frontal_Inf_Tri_L
	Thalamus_R	4.3	507	1.7	-11.9	4.2	Thalamus_R, Thalamus_L
	Parietal_Sup_L	3.9	1255	-21.2	-64.3	45.5	Parietal_Inf_L, Parietal_Sup_L, Angular_L, Occipital_Mid_L
	Fusiform_R	3.8	236	43.0	-18.6	-26.8	Fusiform_R, Temporal_Inf_R
	Temporal_Inf_L	3.7	327	-41.8	-17.8	-28.3	Temporal_Inf_L
	Cingulum_Mid_R	3.4	105	4.6	20.5	31.5	Cingulum_Mid_R, Cingulum_Ant_R
	Temporal_Mid_R	3.3	47	43.7	-65.0	5.7	Temporal_Mid_R
	Supp_Motor_Area_R	3.3	97	9.0	-16.4	73.5	Supp_Motor_Area_R, Precentral_R

[a]Anatomical region where the peak voxel is located using automated anatomical labeling (AAL); [b]The volume in each area is number of voxels multiply by volume in each voxel (provided in cubic millimeters:mm^3); [c]Peak stereotaxic coordinates are reported in Montreal Neurological Institute (MNI) space; [d]Anatomical regions associated with the cluster; Left (L) and Right (R) cortical hemisphere; *number of voxels>1000.

Supplementary Table 4. SBM components showing significant effect of site

	pFWER	*pFDR*	R^2	F(1,163)	Effect size (G)	$CI_{95\%}$
IC 5	<0.0001	<0.0001	0.53	0.06	2.06^{L}	1.61 - 2.43
IC 7	<0.005	<0.005	0.30	38.03	-0.72^{M}	-1.10 - -0.34
IC 15	<0.0001	<0.0001	0.48	2.78	1.97^{L}	1.56 - 2.30
IC 16	<0.0001	<0.0001	0.28	1.27	-1.25^{L}	-1.57 - -0.88
IC 19	<0.0001	<0.0001	0.41	5.07	-1.73^{L}	-2.05 - -1.34

Statistical significance was set to *pFWER<0.05,* Holm-Bonferroni correction. Effect size Cohen's G (^{L}large,^{M}moderate).

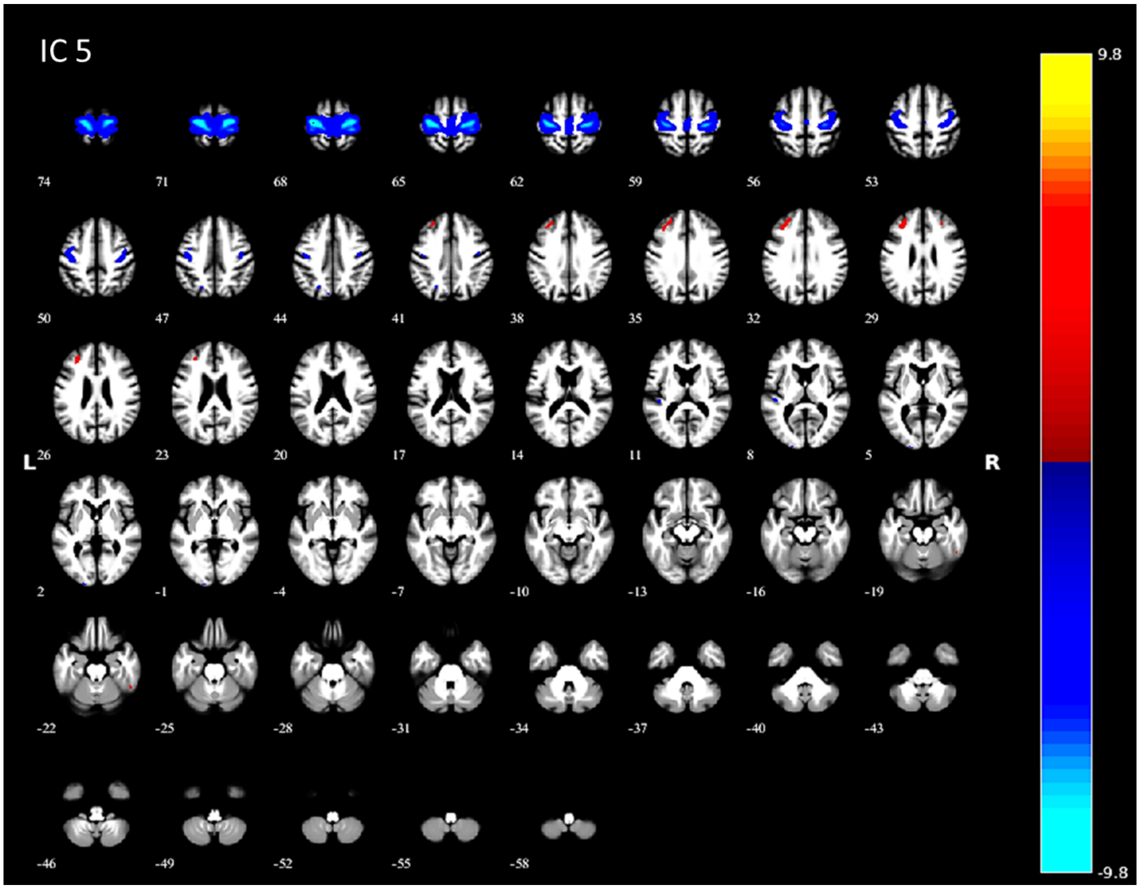

Supplementary Figure 1. Spatial map of IC 5. Light-dark blue colored regions show decreased gray matter in aMCI relative to Controls; Red-yellow colored regions show increased gray matter in aMCI relative to Controls. The color bar shows color mapping for the normalized component weights (Z-scores, thresholded at | 3 |). aMCI, amnestic mild cognitive impairment; IC, Independent Component. Left (L) and Right (R) cortical hemisphere.

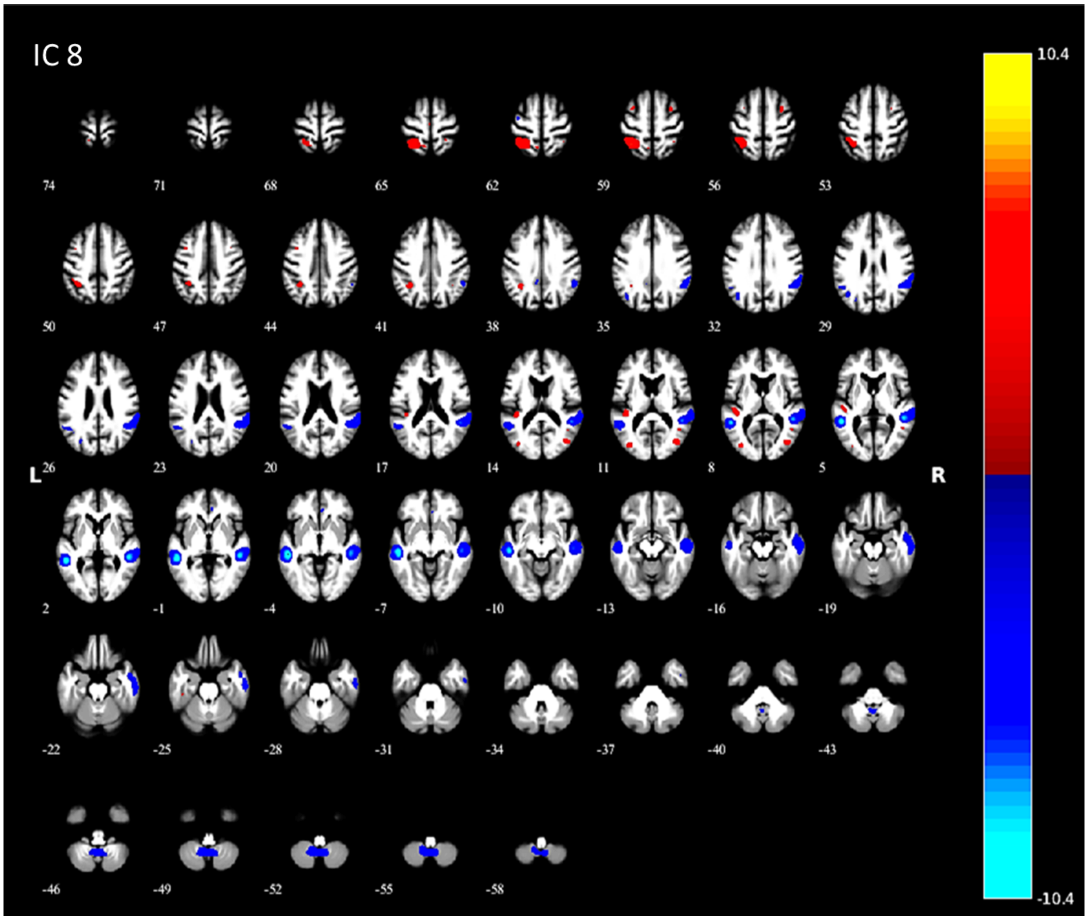

Supplementary Figure 2. Spatial map of IC 8. Light-dark blue colored regions show decreased gray matter in aMCI relative to Controls; Red-yellow colored regions show increased gray matter in aMCI relative to Controls. The color bar shows color mapping for the normalized component weights (Z-scores, thresholded at | 3 |). aMCI, amnestic mild cognitive impairment; IC, Independent Component. Left (L) and Right (R) cortical hemisphere.

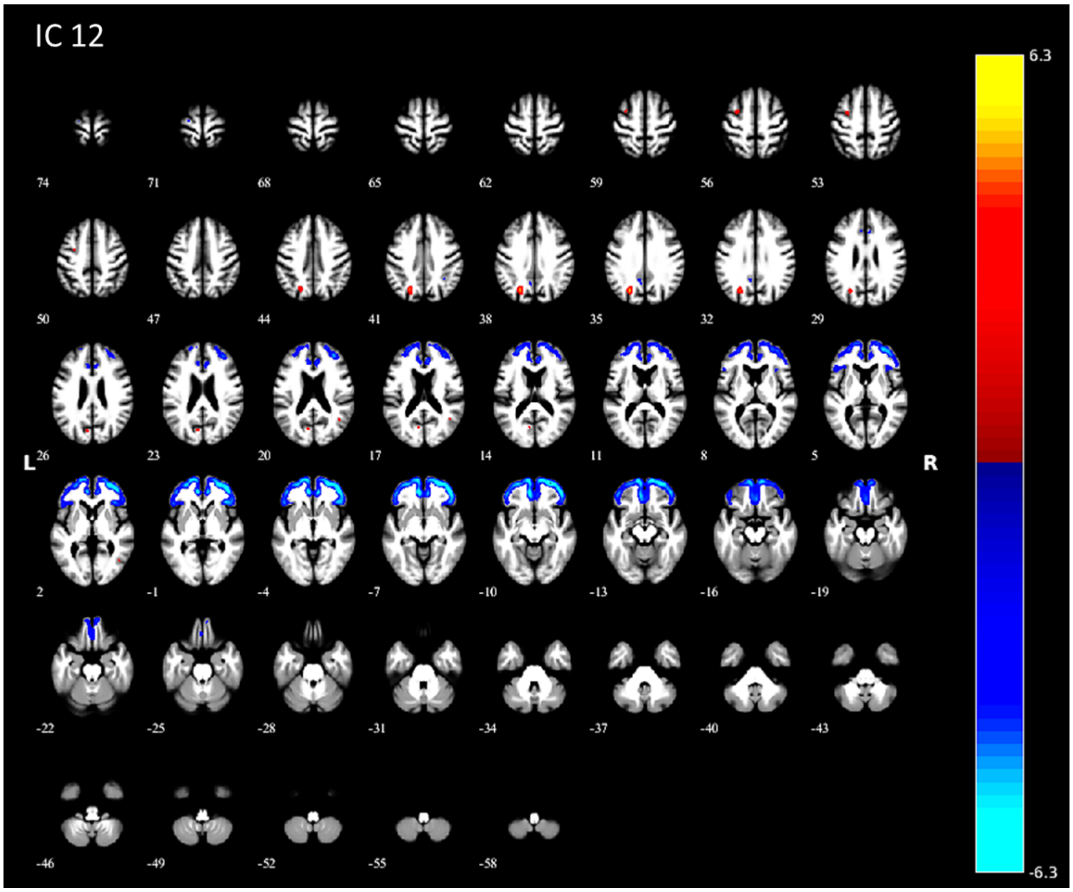

Supplementary Figure 3. Spatial map of IC 12. Light-dark blue colored regions show decreased gray matter in aMCI relative to Controls; Red-yellow colored regions show increased gray matter in aMCI relative to Controls. The color bar shows color mapping for the normalized component weights (Z-scores, thresholded at | 3 |). aMCI, amnestic mild cognitive impairment; IC, Independent Component. Left (L) and Right (R) cortical hemisphere.

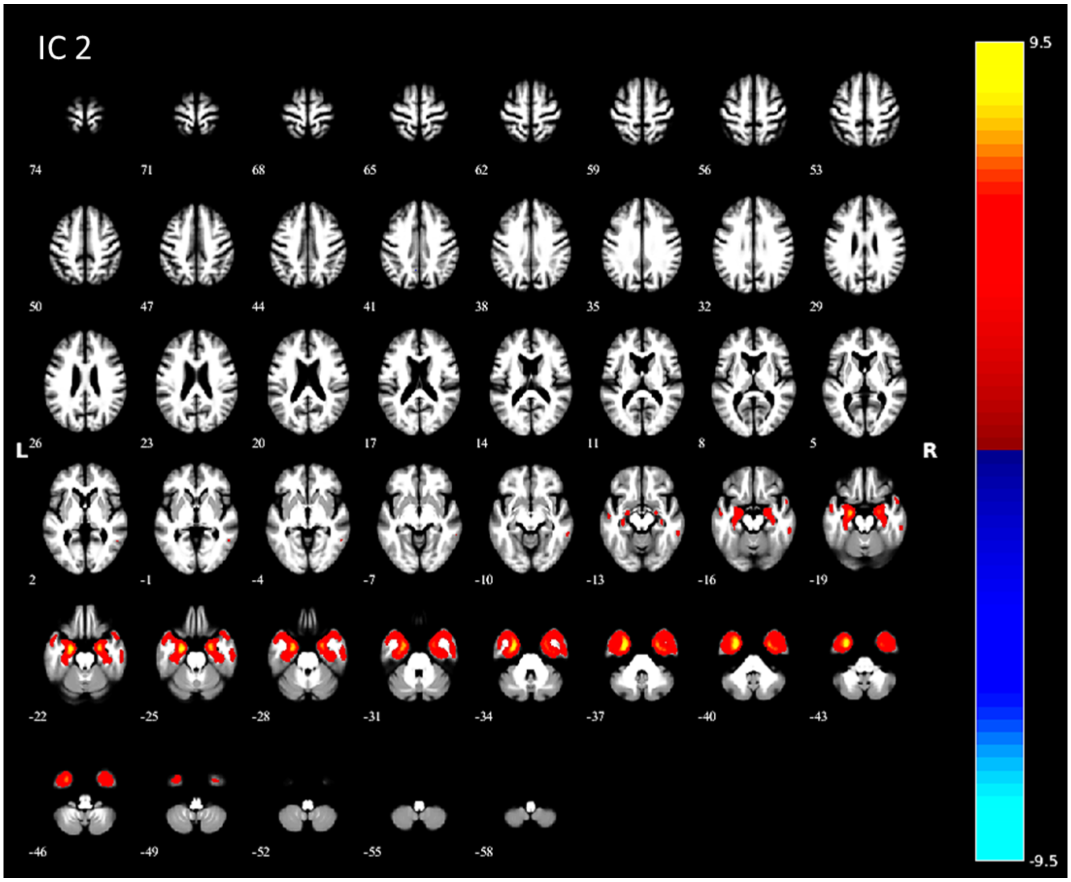

Supplementary Figure 4. Spatial map of IC 2. Red-yellow colored regions show increased gray matter in Controls relative to aMCI (vice versa). The color bar shows color mapping for the normalized component weights (Z-scores, thresholded at | 3 |). aMCI, amnestic mild cognitive impairment; IC, Independent Component. Left (L) and Right (R) cortical hemisphere.
